# LED light can falsify pulse oximetry readings via the stroboscopic effect

**DOI:** 10.1007/s13246-023-01328-2

**Published:** 2023-09-19

**Authors:** Martin Wald, Peter Erwin, Natalee Annon-Eberharter, Tobias Werther

**Affiliations:** 1 Division of Neonatology, Department of Pediatrics and Adolescent Medicine, Paracelsus Medical University, University Hospital Salzburg, Salzburg, Austria; 2 Division of Neonatology, Pediatric Intensive Care and Neuropediatrics, Department of Pediatrics and Adolescent Medicine, Comprehensive Center for Pediatrics, Medical University of Vienna, University Hospital Vienna, Vienna, Austria

**Keywords:** Pulse oximetry, Light-emitting diode, Artifacts, Stroboscopic effect

## Abstract

Because of its simplicity, pulse oximetry plays a ubiquitous role in neonatology. Its measurements are based on the absorption of light by hemoglobin. Ambient light can affect these values, therefore algorithms are designed to compensate for constant ambient light. Modern light-emitting diodes often flicker at a very high frequency. Such flickering ambient light can lead to significant measurement errors in saturation. To present a novel way in which light-emitting diodes influence the function of pulse oximeters and to demonstrate mathematically that a stroboscopic effect may well be responsible for this disturbance. Using publicly available data, a mathematical model of a pulse oximeter with a calibration curve and a proprietary measurement algorithm was created. This was used to simulate saturation measurements in flickering ambient light. To do this, photopletysmograms for red and infrared light at 98% oxygen saturation were mathematically superimposed on the light emission from an examination lamp used in the intensive care unit. From these results, presumable saturation measurements from a pulse oximeter were extrapolated. The light-emitting diodes in the examination lamp flicker at 207 Hz. The pulsating light from the light-emitting diodes causes superimposition of the photoplethysmogram due to the stroboscopic effect. With increasing brightness, the saturation dropped to 85% and the pulse rate to 108 bpm. The pulsed light of light-emitting diodes can distort pulse oximetry measurements. The stroboscopic effect leads to low saturation values, which can lead to the risk of blindness in premature infants due to excessive oxygenation.

## Introduction

It is a well-known phenomenon in neonatal intensive care units that pulse oximeters display saturation and heart rate values, even when not attached to a patient. These measurements are usually expressed as values below 90% saturation. New however, was an event in which an infant after intubation and well-established ventilation, did not according to pulse oximetry readings, achieve oxygenation above 90% saturation. These lower values persisted on the patient monitor (Philips IntelliVue X3, Koninklijke Philips N.V., Amsterdam, Netherlands with Nellcor™ pulse oximetry with Oximax™ technology, Medtronic plc, Dublin, Ireland) despite increasing the fraction of inspired oxygen (FiO_2_) via a closed loop control (SPO_2_C, Fritz Stephan GmbH, Gackenbach, Germany). Surprisingly, an abrupt increase in the recorded saturation occurred when the examination LED (light-emitting diode) lamp (Dräger Variolux®, Drägerwerk AG & Co, Lübeck, Germany) was switched off. Within seconds, the monitor indicated 100% saturation, and the FiO_2_ was reduced to ambient air. Each time the lamp illuminated the infant, a similar drop in oxygen saturation (SpO_2_) could be observed. The saturation sensor was never in the central light cone but was only illuminated by the scattered light of the lamp. The event was reproduced by attaching the sensor of the pulse oximeter to the finger of another subject and placing his finger in the stray light from the LED lamp. The effect disappeared when an opaque shield was placed over the sensor. This indicated that the interference was caused by light and not electromagnetic interference.

For clarification, the occurrence was brought to the attention of all manufacturers involved. Neither the manufacturers, nor a review of scientific literature could provide an explanation for this phenomenon. To gather further information, we repeated the pulse oximeter measurement on adult subjects with similar results. Interestingly, the recordings of the test measurements using a smartphone camera showed black stripes in the video. These stripes disappeared, as did the effects on the saturation measurement, when the examination light was turned off.

Dark bands typically appear in videos illuminated by LED lighting. Here, the camera’s photo sensor captures images during periods when the flickering LED light is in an off portion of its cycle. The image sensor of a smartphone camera exposes pixel by pixel, row by row, in succession (rolling shutter). The pixels that are active while the light is off, record black dots. Depending on the frequencies of the light and the camera, this results in running or stationary black lines. If all pixels of an image sensor are exposed at the same time (global shutter), a flickering of the image occurs because whole images are recorded in darkness.

This phenomenon is a stroboscopic effect. The stroboscopic effect is the apparent slowing down or reversal in direction of periodic motion observed at regular successive time intervals. Classic examples of the stroboscopic effect are old movies in which the wheels of carriages appear to be turning in the wrong direction. The illusion of motion occurs because the shutter speed of the camera and the speed of the movement of the spokes do not match. The stroboscopic effect posed not only problems with the early film industry, but also in radiology [1]. There, the stroboscopic effect resulted from poor synchronization of a rotating grid and the frequency of the alternating current. Today, black bands appear in videos because the flickering of the LED light is not synchronized with the frame rate.

The LEDs and sensors of pulse oximeters also do not light up and measure continuously. These LEDs switch on and off at a high frequency, in our case, with a frequency of 311.25 Hz [2]. Measurements are made selectively in the light and dark phases respectively [2]. This is similar to the frame rate in film. This comparison led to the suggestion that the two observed anomalies are based on similar physical principles.

The purpose of this study is to establish a mathematical model, which supports the clinical observation of an LED light triggering erroneous saturation measurements in a pulse oximeter and, more precisely, to show that this outcome is a direct result of the stroboscopic effect triggered by the light source.

## Methods

Functionality of an LED lamp: LED lamps consist of several light emitting diodes operating on direct current. Continuously operating diodes can age and dim gradually. For this reason, pulse width modulation (PWM) is often used [3–5]. PWM is a technique for controlling the power delivered to electrical devices. In LEDs, this is accomplished by switching the diodes on and off in rapid succession. Modulation also enables LED lamps to be dimmed.

How a pulse oximeter works: A pulse oximeter is based on Beer-Lambert’s law. This states that a light transmitted through a substance is attenuated [6]. The attenuation or absorbance is the result of the thickness of the layer, concentration of the attenuating species, and the extinction coefficient of the substance being transmitted [2, 7]. The volume and concentration of arterial hemoglobin in a tissue increase during systole and decrease during diastole. Correspondingly, the light passing through a tissue decreases during systole and increases during diastole [8]. The resulting curve corresponds to an arterial blood pressure curve mirrored across the time axis.

The arterial blood volume corresponds to only a small fraction of the tissue. Accordingly, most of the extinction remains constant throughout the cardiac cycle. The extinction of light by tissue can thus be divided into a very small pulsatile alternating current (AC) component and a large constant direct current (DC) component [9]. The AC amplitude corresponds to the difference between the maximum and minimum light transmittance. The DC fraction corresponds to the mean light transmission through the tissue [8].

Hemoglobin exhibits characteristic absorbance at particular wavelengths of light, depending on its oxygenation [7]. Therefore, both the AC and DC components of the absorbance at different wavelengths of light vary as a function of oxygenation. The relative magnitude ratios of the amplitudes to each other remain constant regardless of individual factors such as hematocrit, body size, or cardiac output. For this reason, the oxygenation of hemoglobin can be inferred from the ratio of the relative magnitudes of the AC amplitudes of two different wavelengths. Modern pulse oximeters make use of the 660 and 940 nm wavelengths, corresponding to red (RED) and near infrared (IR) regions, respectively [2], in order compute a double ratio (R) of AC to DC components in both these regions. This is defined by:

1 $$R= \frac{{AC}_{RED}}{{DC}_{RED}} / \frac{{AC}_{IR}}{{DC}_{IR}}$$

With this value, the arterial saturation can then be derived using an internal algorithm [10].

The approach of this study was to mathematically simulate this process of determining an arterial saturation under an ambient LED light of varying intensity. Whenever possible, the devices that were in use during the initial observation described in the introduction were used to conduct this study.

### Study design

**Fig. 1:**
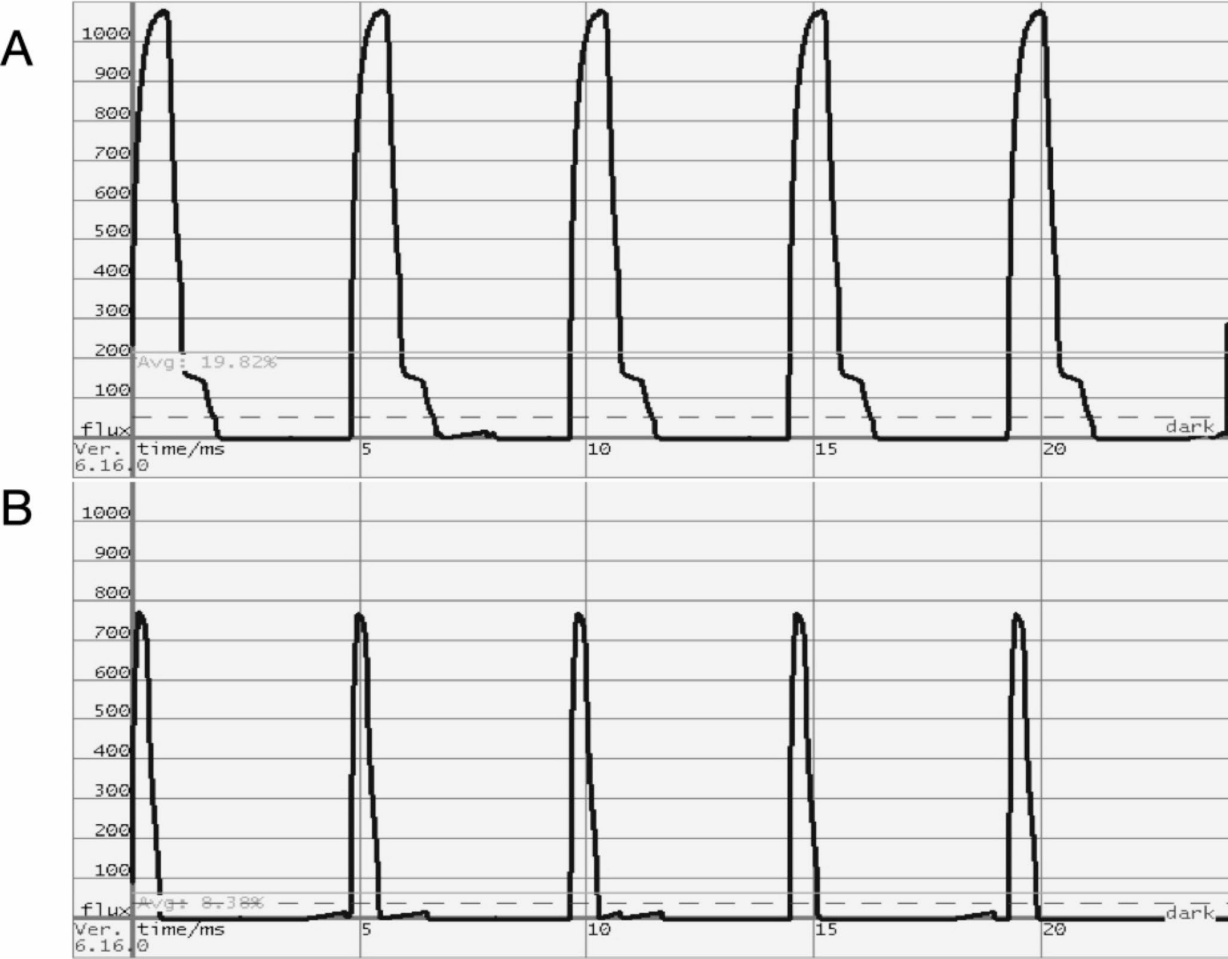
Light emission of the Dräger Variolux® (Drägerwerk AG & Co, Lübeck, Germany) in non-dimmed (**A**) and maximum dimmed (**B**) condition. Light emission over time was 37% in non-dimmed mode and 13% fully dimmed mode

**Fig. 2:**
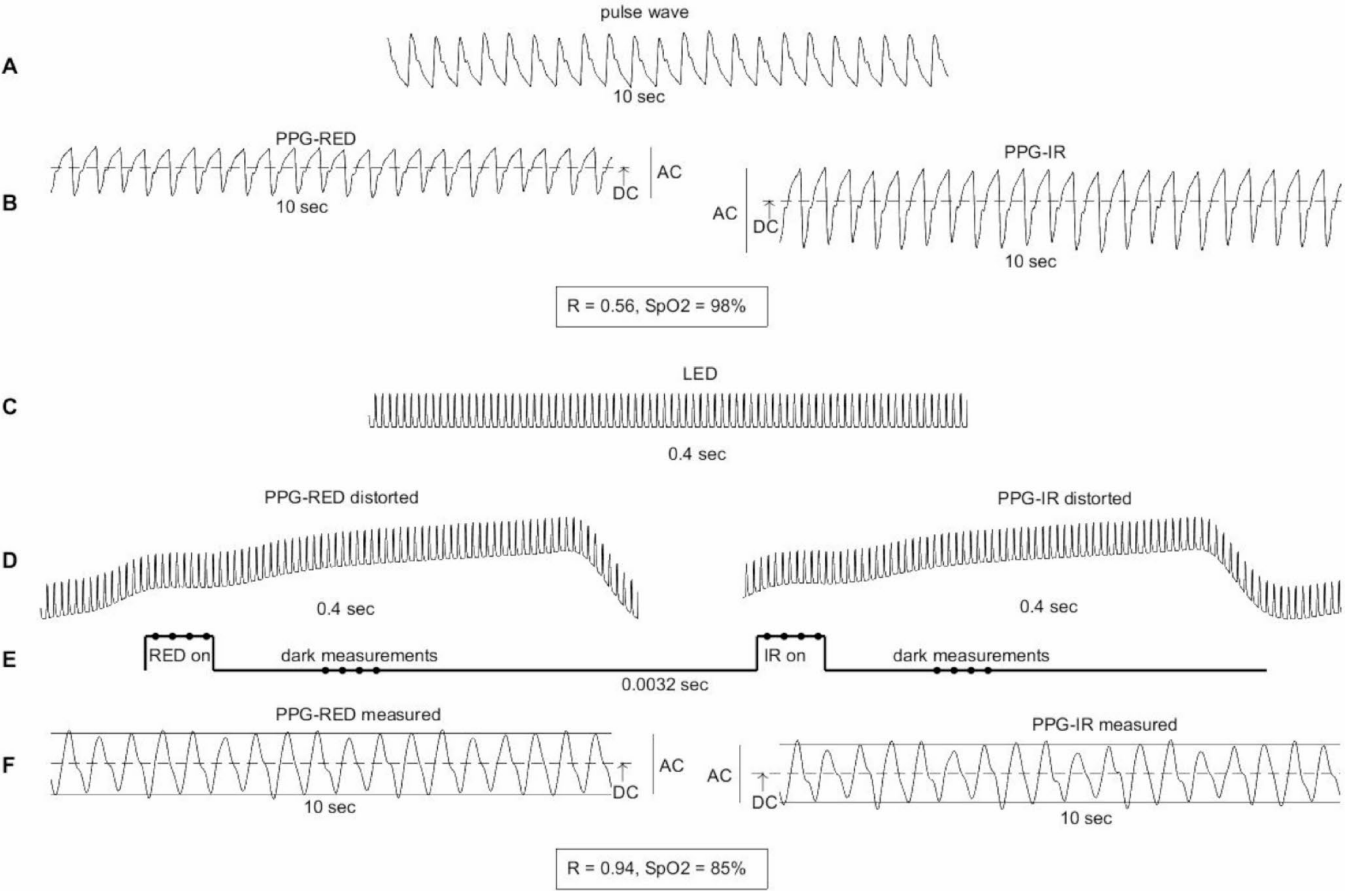
Pulse-oximeter model. (**A**) pulse wave (10 s) derived from an arterial blood pressure signal. (**B**) Photoplethysmogram (PPG) of the red and the infrared (IR) signal with adapted alternating current (AC) and the direct current (DC), representing pulsatile and non-pulsatile regions, respectively. DC values for the red PPGs were set to 20,000 and the infrared PPGs were set to 19,000. The corresponding AC values were specified by 0.5% of the DC values. AC/DC ratio determines the normalized absorbance. The ratio of normalized absorbance between the red and the infrared signal determines the light ratio (R), which specifies the peripheral oxygen saturation by means of a calibration curve. (**C**) LED source with a duration of 0.4 s. (**D**) The LED superimposed to each PPG with a distortion factor of 2000 (approximately one tenth of the DC). (**E**) Model of the pulse oximeter which generates a value of the red PPG and the infrared PPG by averaging four measurements (dots at “RED on” and at “IR on”) and subtracting the average of the consecutive four dark measurements, respectively. The measurements of the red PPG and the infrared PPG are time-shifted and occur once every 0.0032 s, generating PPG signals with a sampling frequency of 311.25 Hz. (**F**) Display of the PPG signals with corresponding light ratio and SpO_2_ value

To determine the LED ambient light, the first step was to determine the frequency of PWM and the light emission of the LED examination lamp. This was done by using the compact flicker degree (CFD) method for the LED examination lamp. The CFD is a unit of measure light flicker in percent, which is the Pythagorean Sum of all frequency-dependent weighted frequency components of the AC part of a light signal. The frequency-dependent weighting is proportional to the frequency-dependent perception sensitivity of the human being [11]. When calculating the CFD, the light emission of the lamp is measured in a standardized manner. The LED examination lamp was measured both in the non-dimmed and in the maximally dimmed state. Both measurement curves are shown in Fig. 1. From the results for the non-dimmed light, digital LED signal with a sampling rate of 100 kHz (kHz) was generated (Fig. 2C).

**Fig. 3:**
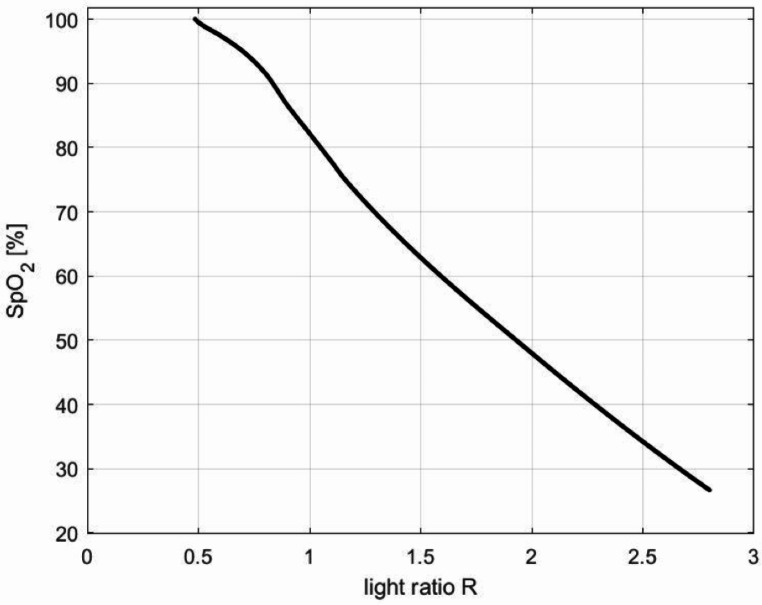
Calibration curve (ratio of normalized absorbance and corresponding oxygen saturation)

In the second step, an artificial calibration curve was created for the pulse oximeter because the calibration curve of the pulse oximeter used was not accessible. For this, the Masimo pulse oximeter curve published by Johnston et al. 2011 was used as a prototype and the fitting requirements outlined by Lakshminrusimha et al. 2015 were considered [12, 13]. In this way, the R-values for a fictitious pulse oximeter could be defined. The artificial calibration curve is shown in Fig. 3.

In the third step, photoplethysmograms (PPG) for RED and IR were generated. To obtain a neonatal pulse wave, an arterial blood pressure signal freely available (https://github.com/jaj42/BP_annotate/blob/master/BP_annotate_GUI/exampleWaveform_1kHz.mat) with a heart rate of 55 bpm was compressed to a frequency of 140 bpm and limited to a length of 12 s. This signal was also upsampled to 100 kHz. By inverting this curve, an artificial PPG curve of a premature infant was created, which is shown in Fig. 2A. This artificial PPG curve was used as a basis for both RED and IR. The AC part was set at 0.5% and the DC part at 99.5% for the RED PPG signal and 0.9% and t 99.1%, respectively for the IR PPG signal. This achieved an R-value of 0.55 and corresponded to an oxygen saturation of 98%, according to the calibration curve. The two PPG curves are shown in Fig. 2B.

In the fourth step, we simulated recording and digital processing of the light interference of the RED and IR PPG curves with the LED light. It was assumed that the recordings of the pulse oximeter are made with a broadband photodetector that does not distinguish between wavelengths and records every 10 microseconds (corresponding to 100 kHz). This included recordings of the 660 nm red light signal and the 940 nm infrared light signal emitted from the pulse oximeter diode. The photodetector of the pulse oximeter first records the activated red emission, followed by a dark measurement (photodetector is active but no light is emitted from the pulse oximeter), followed by a recording of the activated infrared emission and subsequent dark phase. The measurement in the dark phase is used to correct the red (infrared) signal for ambient light. Specifically, ambient light is recorded during the dark measurement and then subtracted from the measurements of the red (infrared) signal. Assuming the ambient light to be constant at least during the measurement cycle, this will eliminate the disturbance of the ambient light in the measurements of the red (infrared) signal. Ambient LED light, on the other hand, is not constant and therefore will not be eliminated by subtraction of the dark measurements, and can subsequently induce a stroboscopic effect (compare Fig. 2D). According to the manufacturer of the device used, the RED-dark-IR-dark cycle is repeated 311.25 times per second (approximately every 3.2 milliseconds corresponding to 320 samples for a sampling rate of 100 kHz) and the pulse oximeter provides samples of the corrected red and infrared recording at this frequency.

In our model, we simulated the LED disturbance by superimposing the LED signal, multiplied by a distortion factor, to the red (infrared) PPG (Fig. 2D). We simulated the RED-dark-IR-dark cycle (duration 3.2 milliseconds, Fig. 2E) as follows:

1. RED (only the distorted RED PPG signal is “active”): starting at 800 microseconds after the beginning of the cycle, four consecutive samples of the RED PPG signal were averaged.
2. The first dark measurements (only the distorted LED signal is “active”) started 250 microseconds later in which four consecutive samples of the distorted LED signal were averaged.
3. Infra-Red (only the distorted IR PPG signal is “active”): starting at 2.4 milliseconds after the beginning of the cycle, four consecutive samples of the IR PPG signal were averaged.
4. The second dark measurements (only the distorted LED signal is “active”) started 250 microseconds later in which four consecutive samples of the distorted LED signal were averaged.
5. By subtracting the first dark measurement from the RED measurement and the second dark measurement from the IR measurement, a RED output sample and an IR output sample were obtained for each cycle. The output PPG curves, now sampled at the cycling frequency of the pulse oximeter algorithm (311.25 Hz), were smoothed by applying a low-pass filter with a cutoff frequency of 5 Hz. Since the stroboscopic effect depends on the sampling rate (Nyquist-Shannon sampling theorem), we tested the mathematical model with a pulse oximeter sampling rate of 300 Hz, slightly deviating from the original sampling rate (311.25 Hz).
6. The R-value of RED to IR light was calculated for the output PPG signals by calculating the AC component (difference between the mean values of the maxima and minima of the PPG signal) and the DC component (mean value of the PPG). Using the calibration curve, the peripheral oxygen saturation under LED distortion could then be determined from the new R-value of RED to IR light (Fig. 2F).
7. Finally, the pulse frequency was determined using the resulting distorted IR signal. For this purpose, wave maxima were searched for at a minimum interval of 0.2 s in the infrared PPG. This corresponded to a maximum detectable pulse rate of 300 beats per minute (bpm).

To simulate different degrees of LED distortion, the distortion factor was varied and steps four to six described above were repeated accordingly. This allowed different degrees of PPG distortion.

All signal-processing calculations were performed using the MATLAB R2018b programming and numerical computation platform (The MathWorks, Inc., Natick, Massachusetts, USA).

## Results

The LED examination lamp used was operated via pulse wave modulation at a frequency of 207 Hz. The CFD was calculated 63.9% in the non-dimmed mode and 80.1% in the fully dimmed mode. This resulted in light emission only 37% of the time in the non-dimmed mode and 13% of the time in the fully dimmed mode. Figure 1A show the measurement results for the non-dimmed and Fig. 1B from maximally dimmed LED light. The LED signal of the non-dimmed light was used for all further calculations.

**Fig. 4:**
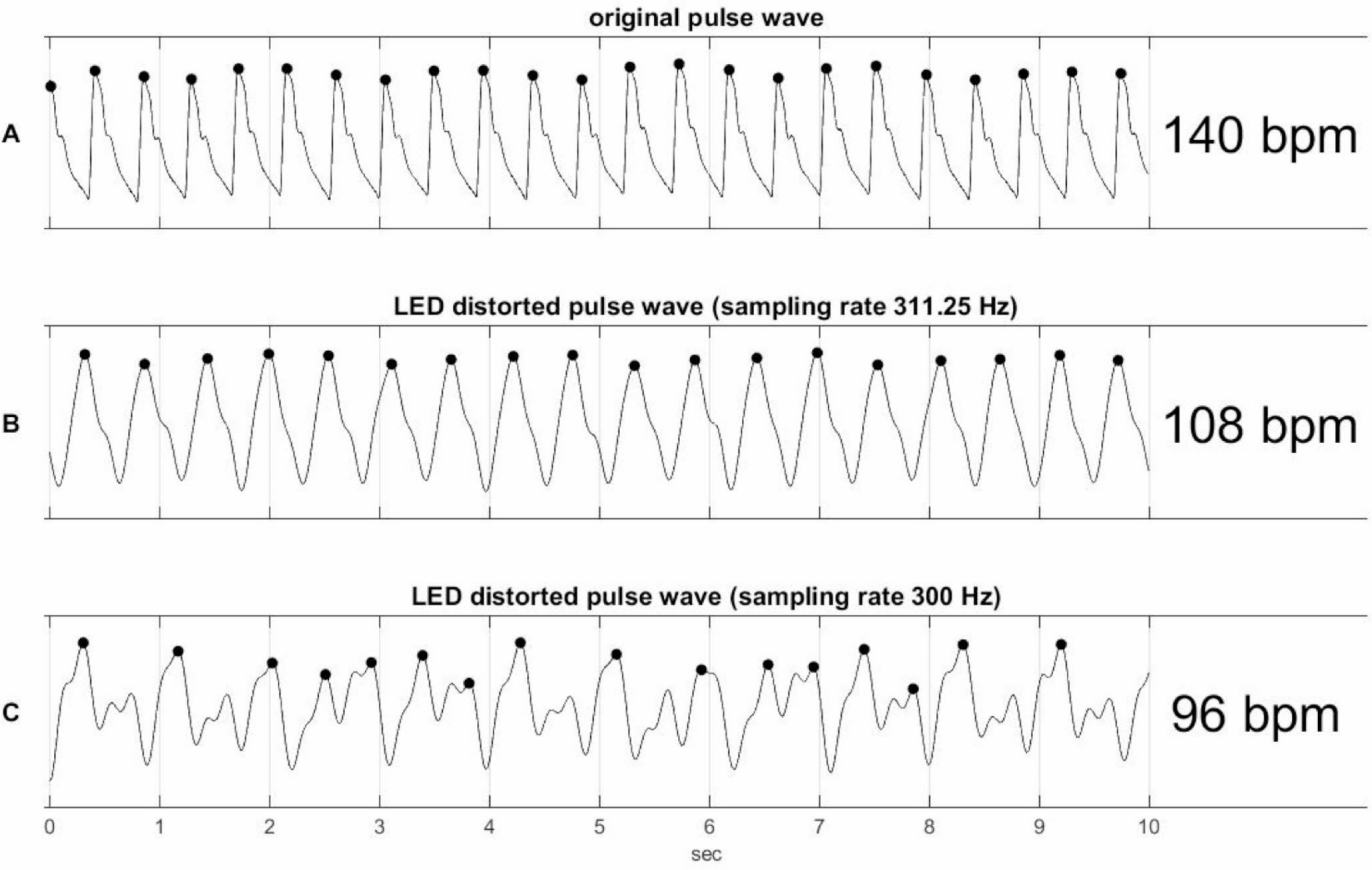
(**A**) Original pulse wave (derived from an arterial pressure signal), (**B**) example of a distorted pulse wave and (**C**) distorted pulse wave with 300 Hz sampling rate. The black dots indicate the maxima of each signal with a minimal distance of 0.2 s

The pulse curve with a pulse rate of 140 bpm was generated from an arterial blood pressure signal (Fig. 4A). Using this pulse curve and a distortion factor of 5000, two distorted pulse waves were derived from the mathematical model of the pulse oximeter with a sampling frequency of 311.25 Hz and 300 Hz, respectively (Fig. 4B and C). The estimated R-values amounted to 0.95 and 0.68 corresponding to SpO2-values of 85% and 96%, respectively. Since a medical doctor would suspect an artefact due to the irregularity of the resulting curve at 300 Hz, only signals with a sampling rate of 311.25 Hz were used in the further course.

**Fig. 5:**
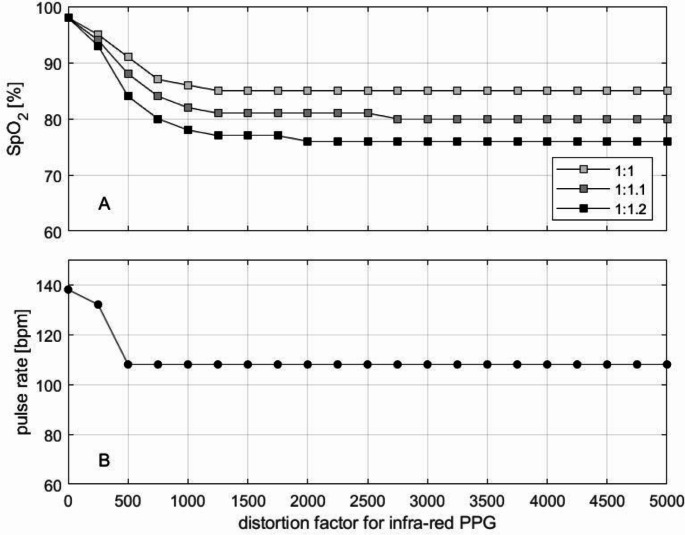
SpO_2_ and pulse rate of distorted photoplethysmogram (PPG) by LED light with increasing distortion factor (x-axis). SpO_2_ with three different distortion ratios for the red to infrared PPG (equal distortion (1:1), distortion augmentation of 10% for the red PPG (1:1.1), distortion augmentation of 20% for the red PPG (1:1.2))

Using a distortion factor of 2000 (approximately one tenth of the DC) for both the red and the infrared signal caused a RED to IR ratio of 0.94, corresponding to 85% saturation. Thereafter, the saturation remained stable at this value. Figure 5A, shows the variation of saturation at a sampling rate of 311.25 Hz for red and infrared light with distortion factors up to one fourth of the original DC component (much larger than the original AC component) and three different distortion ratios for the red and infrared PPG (same distortion, distortion of the red PPG increased by 10% and by 20%). As the distortion of the red light signal increased, the resulting saturation decreased.

The pulse rate also plateaued at 108 bpm despite increasing distortion. Figure 5B, shows the course of the measured pulse frequency as a function of the distortion factor.

## Discussion

The mathematical model we have developed shows that a photoplethysmographic pulse wave can be distorted by the interfering light of an LED lamp with PWM due to the stroboscopic effect. With increased interference from light or increased distortion factor, the saturation decreased to values approximating 85%. Thereafter, it remained unchanged despite further interference by the lamp. An explanation for this plateau may be the pulse oximeter’s use of a broadband photodiode as a detector. The detector assumes that all received light is red light when the red LED light is active, and that all is infrared light when the infrared LED is active. This works if the ambient light can be subtracted from the measured signal. Ambient light is determined in the dark phases when both the red and infrared LEDs are off. The ambient light measured here is then subtracted from the respective subsequent measurement signals of red and infrared. This method works as long as the ambient light is constant and remains within a given threshold. Measuring in excessively bright light overtaxes this system. If the stray light is highly dynamic and alternates between the dark and light phases of the pulse oximeter, it cannot be eliminated by subtraction. This makes the pulse oximeter’s assumption that the measured signal originates only from the sensor LED incorrect. This leads to an interfering signal. If the interfering light signal is sufficiently large, then the changes to the light caused by passing through the tissue becomes a less significant portion of the measured signal. In addition, the interfering signal may affect both wavelengths in a similar way - and may well come close to determining their AC component. (It is not clear that this must always happen - it may have to do with the relation between the PWM frequency and the rate at which the pulse oximeter samples the RED, IR, and dark phases. In our system, the pulse oximeter samples the measured signals at a rate that is almost exactly 1.5 times the frequency of the PWM signals output by the LED, and this may be significant.) Thus, the signals contain relatively little information about the patient’s status. The AC contributions of the RED and IR signals may become nearly equal, and, therefore, the R-value can only be affected by the DC ratios of the two signals, which in our case is 0.95 corresponding to a saturation of approximately 85%. This value can vary from pulse oximeter to pulse oximeter due to the different calibration curves, but the order of magnitude is the same for all devices. This also corresponds exactly to the event that led to the entire investigation. This event also showed that the large amplitude is not a problem for the pulse oximeter.

The distorted pulse wave can also lead to an incorrect pulse rate. The pulse wave displayed on a pulse oximeter is usually derived from the infrared PPG. As previously described, as the ambient light intensity increases, both the PPG of the RED and the IR signal are overlaid by the PWM LED signal, which is represented by an increasing distortion factor. In the end, the pulse oximeter can only process the IR PPG superimposed by the PWM-LED curve. In the presented model with a sampling rate of 311.25 Hz and a distortion factor up to one fourth of the DC component of the infrared PPG, the pulse rate corresponds to a plausible heart rate for neonatal patients. This pulse curve can be interpreted by the pulse oximeter as a valid measurement, particularly when the interference already existed at the beginning of each measurement. The incorrect pulse frequency therefore does not represent a warning signal for an incorrect measurement. When using 300 Hz, the superimposed PPG became so irregular that a pulse frequency but not a credible pulse curve was displayed. We observed more regular output PPG signals while using 414 (twice the LED frequency) and 500 Hz. This emphasizes the relationship between LED frequency and pulse oximeter frequency. A more detailed description of this relationship was beyond the scope of this study.

In theory, LED light does not necessarily cause the interference described here. The phenomenon of a saturation measurement displaying on the monitor, although the pulse oximeter is not attached to a patient, occurs even when no LED light source is present. It can therefore be assumed that such phantom signals can be caused by any flickering light source. Both incandescent bulbs and fluorescent tubes flicker during standard operation with alternating current. Consequently, these light sources can also cause the interference signal we have shown.

The possible detriment of using LED light with PWM in a neonatal intensive care unit goes beyond its interaction with pulse oximeters. It is not yet known how flicker affects the immature brains of premature babies. In adults, flickering light can cause seizures [14]. This usually occurs only at low frequencies around 15 Hz. Frequencies above 80 Hz cause stress. Above a frequency of 200 Hz, the phantom array effect occurs. This is a stroboscopic effect with multiple perceptions of an object moving in this light [15]. The light source we studied flickers at 207 Hz, which corresponds to a frequency that can not only cause stress but also multiple perceptions. There are EU regulations in place for the use of indoor lighting, which prohibit strong flickering light at the frequency of the lamps we tested [16]. However, medical lamps, with their specific requirements such as spot brightness and color fidelity as essential diagnostic features, are excluded from these regulations. This should be fundamentally questioned, as the technical effort to produce flicker-free LED lamps seems manageable [17]. However, efforts should be made to avoid flicker not only on the part of the lamps. It is also possible to produce video cameras and image sensors that minimize the stroboscopic effect caused by LEDs [18]. The question is whether such techniques can also be applied to pulse oximeters.

A limitation of the study is the simplified model used to simulate pulse oximetry measurements. In particular we did not test for different versions of the RED-dark-IR-dark cycle which was beyond the scope of his study. Furthermore, we did not use an original Nellcor calibration curve, but a calibration curve of a Masimo pulse oximeter derived according to Johnston 2011 and Lakshminrusimha 2015 [12, 13], although the measurement is based on a Nellcor algorithm. Since the effect of the stroboscopic LED light on the pulse oximeter wave displayed on the monitor was remarkably close to the results of the theoretical model, it can be assumed that our theoretical model is not oversimplified.

For further research it would be interesting and informative to see what effect on the readings a LED lamp with a different PWM frequency, a pulse oximeter with different sampling rates or a different pulse rate of the patient would have. It would be interesting to see if the pulse oximeter really detects that there is something wrong with the filtered PPG signal if it looks more like Fig. 4C than Fig. 4B. It would also be interesting to see what saturation value the pulse oximeter shows. These questions were outside the scope of the current study.

## Conclusion

The mathematical model we have developed shows that the photoplethysmographic pulse wave can be distorted by the interfering light of an LED lamp with PWM. According to the CFD measurements, the examination lamps in our neonatal unit flicker strongly. Flickering LED lamps are associated with dizziness, headaches, and stress. Here, we highlight a previously undescribed health risk it poses to newborns. LED flickering adversely affects pulse oximeter measurements due to the stroboscopic effect. In our theoretical model, we demonstrated a drop in saturation values to about 85%, depending on the level of interference caused by the lamp. False values pose a risk to accurate clinical treatment and potential outcome. Erroneously low saturation levels can lead to unnecessary increases in oxygen administration, which increases the risk of retinopathy in preterm infants [19]. To minimize inaccuracies in pulse oximeter readings, flicker-free LED lamps should be used in medical settings. On the other hand, manufacturers should be quite capable of producing pulse oximeters that can detect artifacts caused by PWM LEDs.

## Data Availability

All data, formulas and documentation are available upon request. Please contact the corresponding author.
